# Using Health Insurance Network Provider Data and Public Data Sets to Identify SARS-CoV-2 Vaccinators in the USA

**DOI:** 10.3389/fpubh.2020.616140

**Published:** 2021-01-25

**Authors:** John R. Litaker, Naomi Tamez, Wesley Durkalski, Richard Taylor

**Affiliations:** ^1^The Litaker Group, LLC, Austin, TX, United States; ^2^Sendero Health Plans, Inc., Austin, TX, United States; ^3^School of Human Ecology, University of Texas at Austin, Austin, TX, United States

**Keywords:** SARS-CoV-2, COVID-19, vaccination, mass vaccination, Sendero Health Plans, vaccinators

## Abstract

**Objective:** Mass vaccination planning is occurring at all levels of government in advance of regulatory approval and manufacture of a SARS-CoV-2 vaccine for distribution sometime in 2021. We outline a methodology in which both health insurance provider network data and publicly available data sources can be used to identify and plan for SARS-CoV-2 vaccinator capacity at the county level.

**Methods:** Sendero Health Plans, Inc. provider network data, Texas State Board of Pharmacy data, US Census Bureau data, and H1N1 monovalent vaccine data were utilized to identify providers with demonstrated capacity to vaccinate the population in Travis County, Texas to achieve an estimated SARS-CoV-2 herd immunity target of 67%.

**Results:** Within the Sendero network, 2,356 non-pharmacy providers were identified with 788 (33.4%) practicing in primary care and 1,569 (66.6%) practicing as specialists. Of the total, 686 (29.1%) provided at least one immunization between January 1, 2019 and September 30, 2020. There are 300 pharmacies with active licenses in Travis County with 161 (53.7%) classified as community pharmacies. We estimate that 1,707,098 doses of a 2-dose SARS-CoV-2 vaccine series will need to be administered within Travis County, Texas to achieve the estimated 67% herd immunity threshold to disrupt person-to-person transmission of the SARS-CoV-2 virus based on 2020 census data.

**Conclusion:** A community-based health insurance plan can use data from its provider network and public data sources to support the CDC call to action to identify SARS-CoV-2 vaccinators in the community, including physicians, nurse practitioners, physician assistants, and pharmacies in order to provide macro level estimates of SARS-CoV-2 administration and throughput.

## Introduction

The US Centers for Disease Control and Prevention (CDC) released guidance on August 31, 2020 outlining the nationwide process for distributing and administering the SARS-CoV-2 vaccine (1). On September 16, 2020 the CDC released an interim “playbook” to guide jurisdictional operations on vaccine distribution and administration (2). The timeline for implementing this guidance is aggressive and reflects the need to prepare for and enable both logistical and operational components of vaccine distribution in advance of regulatory approval and vaccine manufacture. The logistical and operational components of vaccine distribution are well-established for childhood vaccines; however, these processes have not been applied to vaccine distribution on the scale, magnitude, and timeframe needed to achieve vaccine-induced herd immunity envisioned for the COVID-19 pandemic. Indeed, the closest comparison for an expedited large-scale vaccine distribution network to providers was during the novel H1N1 influenza pandemic of 2009 when 80.1 million doses of the monovalent H1N1 vaccine were distributed nationally, representing a nationwide monovalent vaccine coverage rate of 27.0% (3, 4).

The challenges related to vaccine distribution and administration of the SARS-CoV-2 vaccine should not be underestimated. Among the many challenges are: (1) distribution of vaccine quantities on a scale never attempted in the United States; (2) the likely need for two-dose administration of the vaccine; (3) an estimated coverage rate of 67.0% needed to achieve vaccine-induced herd immunity (which is nearly 2.5 times higher than the monovalent H1N1 vaccine coverage achieved in 2009) (3); and (4) inherent limitations on provider enrollment and capacity. (There are other logistical and operational challenges related to cold chain management and storage, particularly requirements related to ultracold storage at −70°C for the CDC labeled “Vaccine A”; however, cold chain management and storage is not within the scope of this paper.)

This policy paper reviews the challenges related to vaccine distribution and administration with a focus on identifying SARS-CoV-2 vaccinators in the community. Further, it outlines how managed care provider network data from a community-based health insurance plan can be used to assist public health officials to identify existing community providers with a demonstrated capacity to support SARS-CoV-2 mass vaccination activities. Concepts identified in this policy paper will be illustrated using Sendero Health Plans, Inc. (Sendero) provider network data, Texas State Board of Pharmacy data, US Census Bureau data, and H1N1 monovalent vaccine data.

## Background

Implementing a mass vaccination strategy is a complicated process. Currently, state and local health departments across the United States are considering using a combination of open and closed Points of Distribution (POD) sites, mobile immunization teams for vulnerable populations, and private clinics and pharmacies to support mass vaccination activities. With regard to the latter, CDC guidance advises jurisdictions to identify providers in the community who can provide vaccination services when expanded quantities of vaccine are available beyond that required for critical workforce populations (5). Centers for Disease Control and Prevention guidance notes that jurisdictions should establish and build upon existing relationships, including with “health insurance issuers” to identify SARS-CoV-2 vaccinators (2).

## Methodology

**Figure 1:**
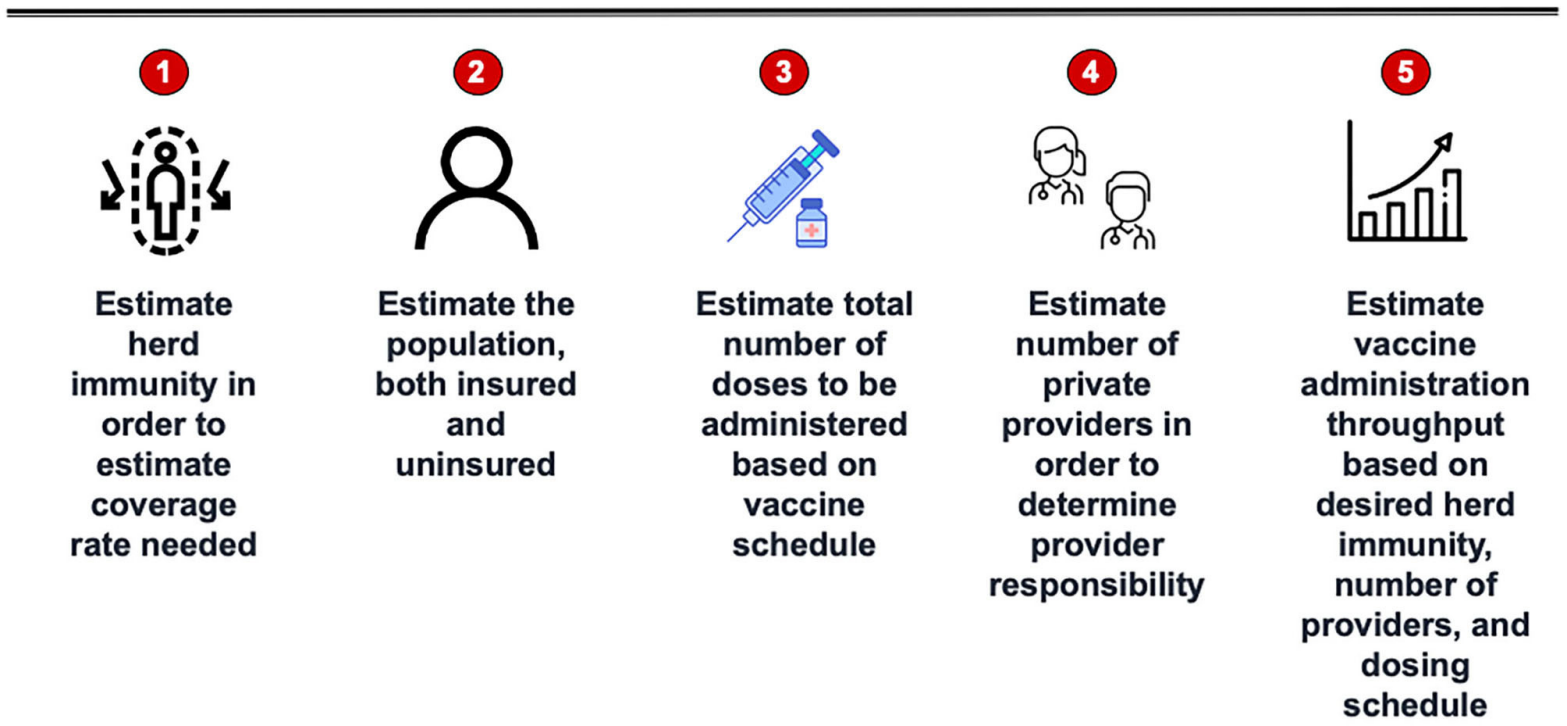
Model to estimate vaccine quantity and providers.

A five step methodology is outlined to estimate quantities of vaccine that a jurisdiction can be expected to provide to achieve herd immunity and to identify potential SARS-CoV-2 vaccinators in the community (see Figure 1). These steps include:

1. Estimating herd immunity
2. Estimating vaccine coverage rates
3. Implementing the SARS-CoV-2 vaccine schedule
4. Identifying providers with demonstrated capacity to administer the SARS-CoV-2 vaccine
5. Estimating vaccine throughput

### Estimating Herd Immunity

Herd immunity is the level of protection against a pathogen that must be attained in the population to disrupt person-to-person transmission of a virus (6). It is a population health metric that is often associated with success or failure of a vaccination campaign. Reaching the herd immunity threshold therefore allows for protection against the virus at the population level, even for those who are unable to be immunized (e.g., because of medical contraindications or because they are immunocompromised). One estimate of herd immunity for the SARS-CoV-2 virus is 67.0% (6).

From a technical perspective herd immunity is the sum of naturally acquired immunity and vaccine induced immunity in a given population. The level of vaccine-induced immunity required for a particular population is therefore dependent on the level of naturally acquired immunity achieved during community transmission of the virus. For this paper we will assume that herd immunity and vaccine coverage for SARS-CoV-2 are equal because, while rigorous in its estimation, a precise herd immunity threshold cannot be calculated until the basic reproduction number (R_0_) is confirmed.

### Estimating Vaccine Coverage Rates

Estimating vaccine coverage is a function of both past experience and the likely expectations and assumptions for the future. The only comparison example of a recent large scale, nationwide, mass vaccination campaign using the private provider network occurred during the 2009/10 H1N1 pandemic. As such, coverage rates during that incident can provide a baseline expectation of SARS-CoV-2 vaccine coverage. Additionally, assumptions about the current pandemic can provide a guide for expected coverage. For example, the demand for the SARS-CoV-2 vaccine will likely be high because of the reported morbidity and mortality associated with COVID-19 and because elected leaders and health officials have noted that herd immunity is necessary before economic and social activities can return to pre-COVID-19 levels.

Nationally, the CDC estimated that 80.1 million (27.0%) persons aged 6 months and older received the monovalent H1N1 vaccine in 2009/10 (3). Coverage varied across age and risk factor cohorts with persons aged 25–64 years having the lowest rate of vaccine coverage at 16.7% (17.8 million doses) and children aged 6 months−17 years having the highest rate of vaccine coverage at 40.2% (29.1 million doses). At the county level, the Texas Department of State Health Services distributed 326,095 doses of the monovalent H1N1 vaccine to providers in Travis County, Texas through August 3, 2010 (7). Based on the 2010 Travis County population of 1,024,266 (8) the estimated monovalent H1N1 vaccine coverage rate for provider administered vaccine was 31.8%. An additional 32,300 doses were distributed to the local health department in Austin, Texas (9). Vaccine distribution data provided by the Texas Department of State Health Services does not account for unused, spoiled, expired, or wasted H1N1 vaccine. Therefore, the numerator is more accurately a measure of distributed vaccine, not administered vaccine, and may serve to overestimate vaccine coverage in the population when distribution data is used as a proxy for vaccine administration.

### Implementing the SARS-CoV-2 Vaccine Schedule

Current CDC planning guidance outlines two potential vaccine scenarios. Both scenarios involve vaccines that have a 2-dose schedule (10). Vaccine Scenario A denotes a 2-dose series to be administered 21 days apart using an ultracold vaccine that requires storage at −70°C, thawing, and reconstitution before administration. Vaccine Scenario B denotes a 2-dose series to be administered 28 days apart with refrigeration between 2 and 8°C, no on-site mixing, and administration within 7–14 days of refrigeration. No 1-dose scenario has been outlined by the CDC. Distribution and administration of Vaccine A will likely be limited to healthcare professionals, essential workers, and long-term care facility staff and residents (10).

From a practical perspective, this means that each person will receive two-doses of the SARS-CoV-2 vaccine. This doubles the volume of vaccine that must be distributed, stored, and administered when compared to the H1N1 single dose monovalent vaccine. The increased quantity of SARS-CoV-2 vaccine will have storage implications at the provider level and will require appropriate staffing levels to maintain vaccine administration throughput. In addition, the primary and secondary dose must be matched by vaccine manufacturer, presenting additional logistical requirements for ordering and storage.

### Identifying COVID-19 Vaccination Providers

Jurisdictions are advised to develop a network of trained, competent, and accessible providers as part of the overall mass vaccination strategy. Centers for Disease Control and Prevention guidance advises jurisdictions to recruit—among others—doctor's offices, pharmacies, and occupational health settings of large employers (to develop closed PODs). This is largely a renewal of the successful strategy used during H1N1 to expand the number and locations of providers to vaccinate as many people as possible. Explicit in this guidance is that jurisdictions should build upon relationships with existing partners, including health insurance companies, to identify potential SARS-CoV-2 vaccinators. To assist in this call to action, Sendero used its provider network to identify potential SARS-CoV-2 vaccinators within Travis County, Texas. Providers were identified as follows:

1. We identified all providers and pharmacies in the Sendero provider network who are eligible to submit a claim for vaccine administration.
  - Providers included doctors of medicine, doctors of osteopath, nurse practitioners, and physician assistants.
  - Providers were further classified as either a specialist or a primary care provider based on pre-identified designations.
2. Of the providers identified in (1) we queried the Sendero claims database to determine how may of these providers administered at least one vaccine based on CPT codes 90460, 90461, 90471, 90472, 90473, 90474.
3. Pharmacies were identified based on Texas State Board of Pharmacy Data. Pharmacies with active licenses were reviewed to determine if they are open to the public for retail services and prescriptions (i.e., community pharmacies). Establishments identified as a community pharmacy were queried in the Sendero claims database to determine if they administered at least one vaccine based on a National Drug Code (NDC) for any vaccine.
4. The date of service for all Sendero data queries was from January 1, 2019 through September 30, 2020.

For data reporting, some data are stratified by individuals with health insurance and without health insurance. Stratifying by health insurance coverage status is designed to more accurately represent likely throughput by provider type. Indiviudals with health insurance are more likely to have a medical home and to use procedures associated with their medical home to obtain the SARS-CoV-2 vaccine when available. Those without health insurance typically do not have a medical home and are likely to utilize public health services to obtain the SARS-CoV-2 vaccine. The SARS-CoV-2 vaccine will be free for consumers at the point of service regardless of health insurance status or place of service. Providers in Texas will be allowed to charge an administration fee based on the state Medicaid schedule.

A total of 2,356 non-pharmacy providers were identified in the Sendero network (see Table 1) Primary care providers represented 787 (33.4%) of those identified and specialists represented 1,569 (66.6%). Among the primary care providers, 538 (68.4%) were designated as vaccinators based on submitting at least one claim for any type of immunization during the study period. Among specialists 148 (9.4%) were similarly designated as vaccinators. In total, 686 (29.1%) Sendero providers are classified as vaccinators while 1,670 (70.9%) are classified as non-vaccinators. Table 2 shows the number of primary care providers who provided at least one immunization during the study period stratified by degree type.

**Table 1:** Sendero provider network of physicians, nurse practitioners, and physician assistants stratified by either specialist or primary care provider type and whether the provider administered at least one vaccine from January 1, 2019 to September 30, 2020.

**Provider type**	**Immunizer**	**Non-immunizer**	**Total**
Primary care provider^*^	538 (68.4%)	249 (31.6%)	787
Specialist	148 (9.4%)	1,421 (90.6%)	1,569
Total	686 (29.1%)	1,670 (70.9%)	2,356

* *Primary care provider include doctors of medicine, doctors of osteopath, nurse practitioners, and physician's assistants*.

**Table 2:** Sendero provider network for physicians, nurse practitioners, and physician assistants stratified by degree type and whether the provider administered at least one vaccine from January 1, 2019 to September 30, 2020.

**Degree**	**Immunizer**	**Non-immunizer**	**Total**
DO	51	19	70
MD	420	96	516
NP	52	106	158
PA	16	28	44
Total	539^*^	249	788

*DO, Doctor of Osteopath; MD, Medical Doctor; NP, Nurse Practitioner; PA, Physician's Assistant*.

* *The primary care provider immunizer total in Table 1 (n = 538) does not match the primary care provider immunizer total in Table 2 (n = 539) because one provider reports two degrees (NP and PA) in the Sendero Network Master Provider List*.

The Texas State Board of Pharmacy reports 300 active, licensed pharmacies in Travis County (11). A review of this data indicates that slightly more than half (*n* = 161; 53.7%) of these licensed pharmacies are open to the public for the purposes of dispensing medicaitons and providing pharmaceutical care. The remaining 139 pharmacies include specialty, compounding, hospital, and government pharmacies, all of whom do not dispense medications directly to the public. Of the 161 community pharmacies in Travis County, 93 (57.8%) administered at least one vaccine to a Sendero member during the observation period, with 87 of these pharmacies classified as a chain community pharmacy (i.e., CVS/pharmacy, Costco, HEB, Randall's, Walgreen's, Walmart, and Sam's Club). Six independent community pharmacies in Travis County dispensed at least one medication to a Sendero member during the study period.

### Estimating Vaccine Throughput

Using the methodology outlined above vaccine throughput by potential SARS-CoV-2 vaccinators can be estimated. The CDC Vaccine Scenario B is used in these estimates because it more accurately represents the technical specifications of the vaccine likely to be distributed to providers and pharmacies. Table 3 outlines throughput using the 686 providers identified by Sendero data using different levels of immunity within the community for those with health insurance. Tables 4 and 5 outline throughput using public health operations (i.e., PODs or mobile vaccination teams) for those without health insurance. Throughput for pharmacies is not included as we do not have enough data to make meaningful estimates; however, it is likely that community pharmacies, particularly chain community pharmacies, will have capacity and will participate as SARS-CoV-2 vaccinators.

**Table 3:** Estimated number of doses of the SARS-CoV-2 vaccine to be administered to persons with health insurance and the mean number of doses to be administered over a 4 week period by providers who have a demonstrated capacity to provide vaccines based on data analysis of the Sendero provider network for five different levels of immunity that could be achieved in the community.

	**Level of immunity to be achieved in the community**				
**Variable**	**40%**	**50%**	**60%**	67%^*^	**70%**
A. 2020 estimated population for Travis County Texas	1,273,954	1,273,954	1,273,954	1,273,954	1,273,954
B. Proportion of people without health insurance	14.8%	14.8%	14.8%	14.8%	14.8%
C. Number of people without health insurance	188,545	188,545	188,545	188,545	188,545
D. Number of people with health insurance	1,085,409	1,085,409	1,085,409	1,085,409	1,085,409
E. Proportion of people to obtain vaccine based on desired level of herd immunity	434,164	542,704	651,245	727,224	759,786
F. Vaccine schedule (doses)	2	2	2	2	2
G. Total number of doses to be administered to people with health insurance	868,327	1,085,409	1,302,491	1,454,448	1,519,572
H. Number of vaccinators (physician, nurse practitioner, physician's assistant)	686	686	686	686	686
I. Mean number of doses to be administered by each vaccinator to meet desired level of herd immunity for people with health insurance	1,266	1,582	1,899	2,120	2,215
J. Mean number of doses to be administered in Round 1	633	791	949	1,060	1,108
K. Mean number of doses to be administered in Round 2	633	791	949	1,060	1,108
L. Mean number of doses administered per hour in each round	4	5	6	7	7

* *67% is the estimated herd immunity level for the SARS-CoV-2 virus*.

*A. Based on US Census Data; B. Based on US Census Data; C. Calculated as the product of (A) times (B); D. Calculated as the difference of (A) minus (C); E. Calculated as the product of (D) times the desired level of herd immunity to be achieved in the community; F. Based on CDC guidance; G. Calculated as the product of (E) times (F); H. Calculated based on the number of doctors, nurse practitioners, and physician assistants who administered at least one vaccine to Sendero members between January 1, 2019 and September 30, 2020; I. Calculated as the quotient of (G) divided by (H); J. The mean number of doses to be administered in Round 1 is the quotient of (I) divided by two; K. The mean number of doses to be administered in Round 2 is the quotient of (I) divided by two; L. Each round is allocated 160 h (four weeks) for vaccine administration. The mean number of doses administered per hour is the quotient of (J) or (K) divided by 160*.

**Table 4:** Estimated number of doses of the SARS-CoV-2 vaccine to be administered to persons without health insurance for five different levels of immunity that could be achieved in the community.

	**Level of immunity to be achieved in the community**				
**Variable**	**40%**	**50%**	**60%**	67%^*^	**70%**
A. Number of people without health insurance	188,545	188,545	188,545	188,545	188,545
B. Proportion of people to obtain vaccine based on desired level of herd immunity	75,418	94,273	113,127	126,325	131,982
C. Vaccine schedule (doses)	2	2	2	2	2
D. Total number of doses to be administered to people without health insurance	150,836	188,545	226,254	252,650	263,963

* *67% is the estimated herd immunity level for the SARS-CoV-2 virus*.

*A. Calculated from Table 3 line (C); B. Calculated at the product of (A) times the desired level of herd immunity to be achieved in the community; C. Based on CDC guidance; D. Calculated as the product of (B) times (C)*.

**Table 5:** Estimated number of doses of the SARS-CoV-2 vaccine to be administered to persons without health insurance and the mean number of doses to be administered over a 4 week period by Points of Distribution sites (*N* = 3) for five different levels of immunity that could be achieved in the community.

	**Level of immunity to be achieved in the community**				
**Variable**	**40%**	**50%**	**60%**	67%^*^	**70%**
A. Total number of doses to be administered to people without health insurance	150,836	188,545	226,254	252,650	263,963
B. Number of POD	3	3	3	3	3
C. Mean number of doses to be administered by each POD to meet desired level of herd immunity for people without health insurance	50,279	62,848	75,418	84,217	87,988
D. Mean number of doses to be administered in Round 1	25,139	31,424	37,709	42,108	43,994
E. Mean number of doses to be administered in Round 2	25,139	31,424	37,709	42,108	43,994
F. Mean number of doses administered per hour in each POD	157	196	236	263	275

* *67% is the estimated herd immunity level for the SARS-CoV-2 virus*.

*A. Calculated from Table 4 line (D); B. Estimated number of PODs that could be established; C. Calculated as the quotient of (A) divided by (B); D. The mean number of doses in to be administered in Round 1 is the quotient of (C) divided by two; E. The mean number of doses to be administered in Round 2 is the quotient of (C) divided by two; F. Each round is allocated 160 h (four weeks) for vaccine administration. The mean number of doses administered per hour is the quotient of (D) or (E) divided by 160 h*.

## Discussion

One or more SARS-CoV-2 vaccines are expected to be approved by regulatory authorities with subsequent manufacturing and distribution in 2021. Centers for Disease Control and Prevention guidance lays out a process for these activities. Guidance documents note that successful implementation of a mass vaccination campaign will be highly dependent on identifying SARS-CoV-2 vaccinator capacity in the community and that “if a jurisdiction has a good understanding of its (SARS-CoV-2) vaccination providers and locations and their vaccine administration capacities, then planners can generate rough estimates of (SARS-CoV-2) vaccination capacity in their jurisdiction (2).”

Partnering with a health insurance company that provides coverage within the jurisdiction of interest can help identify vaccinator capacity. In the methodology outlined above, knowing the types of providers in the community and being able to validate that they have demonstrated capacity based on claims data can assist local officials in understanding who within the community is likely to contribute to mass vaccination activities.

Having access to data from a past pandemic response can also provide a guide to community capacity. In 2009/10 326,095 doses of the monovalent H1N1 vaccine were distributed to providers in Travis County, Texas achieving a vaccine coverage rate of 31.8%. For the current pandemic, 1,454,448 doses for those with health insurance and 252,650 doses for those without health insurance are estimated to be administered to achieve a SARS-CoV-2 herd immunity rate of 67% using a 2-does vaccine schedule. For those with health insurance, this represents nearly four times the quantity of vaccine distributed a decade ago. This is an enormous increase in volume and represents a seismic shift in daily operations for private providers. While the overall vaccination period will continue for many months, once the first dose is provided to a group of individuals, the second dose will need to be provided 28-days apart, representing a minimum 8 week period from start to finish for any vaccine cohort. Indeed, the increase in volume represents over 1 million additional doses to be administered via private providers in Travis County, Texas as compared to 10 years ago.

Some of these doses can also be administered by pharmacies, of which there are 161 commmunity pharmacies in Travis County, Texas. The remaining 139 include compounding, specialty, hospital, and governmental pharmacies. Sendero members preferentially used chain community pharmacies to obtain vaccines during the study period (*n* = 87; 93.5%). Such utilization patterns may reflect ease of access, geographical preference, population density, and general changes in the marketplace that tend to favor chain community pharmacies over independent community pharmacies (12). A detailed analysis of Sendero member pharmacy preference to obtain a vaccine has not been conducted. However, there is general recognition that both independent and chain community pharmacies play a role in supporting public health services like immunizations (12).

It is also important to remember that providers with demonstrated capacity are those who have evidence of vaccine administration, are able to manage the cold-chain storage of vaccines in their clinic or pharmacy, and have the administrative processes and staff in place to record vaccination activity in state immunization registries. State level guidance that suggests vaccinator surge capacity is simply a magnitude of order increase above current vaccine for children providers is naïve and should be viewed with caution as such estimates do not account for cold chain storage capacity or demonstrated experience in vaccine administration. Realistic guidance will, at a minimum, consider the five steps outlined above.

In addition, many potential vaccinators are already at capacity for conducting routine business. While most will naturally want to do their part, it is not possible to estimate the extent of surge capacity for individual clinical practices or pharmacies. The questions then must be asked: (1) how and where will additional staff be recruited to support surge capacity for SARS-CoV-2 vaccination?; (2) what are the plans to train allied health providers to serve as SARS-CoV-2 vaccinators beyond the already established local community capacity?; and (3) what plans and support are available to provide increased vaccine storage capacity at individual clinics or pharmacies to accommodate increased vaccine volume?

Now is the time to prepare the infrastructure and operational activities for SARS-CoV-2 vaccination. The methodology outlined above provides a macro level estimate of vaccinator capacity, the number of doses to be administred, and throughput based on different delivery methods. At the macro level, the stakes are clear—over one million doses of vaccine will need to be administerd in Travis County, Texas in a short period of time in order to disrupt person-to-person transmission of this virus.

## Limitations

This study has several limitations. Firstly, while our provider network data indicate which provider has administered a vaccine between January 1, 2019 and September 30, 2020, we do not know provider vaccine storage capacity, workforce capacity to administer the vaccine, or administrative staff capacity to record vaccine data in state immunization databases as required by the CDC; any limitations in these variables will likely reduce throughput. Secondly, the Sendero provider network does not represent all physicians within Travis County, so it is possible that there is additional SARS-CoV-2 vaccination capacity unaccounted for in this study; however, it would be naïve to think that all currently licensed providers will become SARS-CoV-2 vaccinators, particularly those who do not typically provide vaccines. Thirdly, the data in this paper estimates capacity based on assumed business practices. However, such capacity is subject to change as has been demonstrated by medical office closures for non-emergent procedures during the pandemic. Finally, calculations are based on the analysis using structures applied to a specific country and local jurisdiction within that country.

This study does not address vaccine efficacy. Such information continues to be released as vaccine candidates progress through clinical trial and regulatory processes. Furthermore, this model does not consider infection-induced herd immunity, due to surveillance variability at the national, regional, and local level. Therefore, it is possible that persons who experience asymptomatic disease may choose to receive the vaccine. That said, infection-induced immunity will contribute, along with vaccine-induced immunity, to overall herd immunity within the community.

This study also does not address vulnerability characteristics, social dynamics, and inequity associated with vaccine uptake. Our focus was to create a method to identify likely sources of vaccinator capacity with demonstrated experience to vaccinate the population of a large urban center in the United States. Future work is needed to better understand the dynamics associated with vaccine uptake across the population spectrum within a large urban center in the United States.

## Conclusion

A community-based health insurance plan can use data from its provider network and public data sources to support the CDC call to identify SARS-CoV-2 vaccinator capacity in the community, including physicians, nurse practitioners, physician assistants, and pharmacies. A model is proposed that can be used to develop population estimates of expected quantities of the SARS-CoV-2 vaccine to be received, distributed, and administered at the macro level to achieve different levels of vaccine-induced immunity. This model illustrates the importance of data on the operational and logistical components in preparation of the SARS-CoV-2 mass vaccination campaign.

## Data Availability Statement

The raw data supporting the conclusions of this article will be made available by the authors, without undue reservation.

## Ethics Statement

This project is exempt from Institutional Review Board approval because it does not report on personal health information of Sendero members.

## Author Contributions

JL, RT, NT, and WD substantial contribution to study conception. JL, RT, and NT design of the work, data acquisition or analysis, and interpretation of data. JL and RT drafted work or substantially revised it. All authors contributed to the article and approved the submitted version.

## Conflict of Interest

WD is employed by Sendero Health Plans. The remaining authors are contractors with Sendero Health Plans. All authors declare that the research was conducted in the absence of any commercial or financial relationships that could be construed as a potential conflict of interest.

## Abbreviations

CDC: Centers for Disease Control and Prevention
US: COVID-19, Corona Virus Disease, 2019
CPT: Current Procedural Terminology
H1N1: Hemagglutinin Type 1 and Neuraminidase Type 1 influenza straing
NDC: National Drug Code
POD: Point of Distribution
SARS-CoV-2: Severe acute respiratory syndrome coronavirus 2.
